# Overcoming the cardiac toxicities of cancer therapy immune checkpoint inhibitors

**DOI:** 10.3389/fonc.2022.940127

**Published:** 2022-09-16

**Authors:** Omoruyi Credit Irabor, Nicolas Nelson, Yash Shah, Muneeb Khan Niazi, Spencer Poiset, Eugene Storozynsky, Dinender K. Singla, Douglas Craig Hooper, Bo Lu

**Affiliations:** ^1^ Department of Radiation Oncology, Sidney Kimmel Cancer Center, Philadelphia, PA, United States; ^2^ Department of Radiation Oncology, Thomas Jefferson University, Philadelphia, PA, United States; ^3^ Sidney Kimmel Medical College (SKMC), Philadelphia, PA, United States; ^4^ Division of Cardiology, Thomas Jefferson University Hospital, Philadelphia, PA, United States; ^5^ Division of Metabolic and Cardiovascular Sciences, Burnett School of Biomedical Sciences, College of Medicine, University of Central Florida, Orlando, FL, United States; ^6^ Department of Pharmacology, Physiology, and Cancer Biology, Thomas Jefferson University, Philadelphia, PA, United States

**Keywords:** immune checkpoint inhibitor (ICI), Cardiotoxic adverse effect, immunotherapy, anti PD-1 antibodies, anti CTLA-4 antibodies, anti PD-L1 therapy, Cardiotoxicities

## Abstract

Immune checkpoint inhibitors (ICIs) have led recent advances in the field of cancer immunotherapy improving overall survival in multiple malignancies with abysmal prognoses prior to their introduction. The remarkable efficacy of ICIs is however limited by their potential for systemic and organ specific immune-related adverse events (irAEs), most of which present with mild to moderate symptoms that can resolve spontaneously, with discontinuation of therapy or glucocorticoid therapy. Cardiac irAEs however are potentially fatal. The understanding of autoimmune cardiotoxicity remains limited due to its rareness. In this paper, we provide an updated review of the literature on the pathologic mechanisms, diagnosis, and management of autoimmune cardiotoxicity resulting from ICIs and their combinations and provide perspective on potential strategies and ongoing research developments to prevent and mitigate their occurrence.

## 1 Introduction

In the past few decades, advances in cancer immunotherapy have revolutionized the management of metastatic and advanced-stage malignancies, improving survival in multiple cancers with abysmal prognoses prior to their introduction. On the frontline of these advances are the immune checkpoint inhibitors (ICIs), known to target immune checkpoints, which are critical immune system regulators that can dampen an immune response to a stimulus such as an infection. These inhibitory effects are essential to maintain self-tolerance and prevent over activity of the immune cells. However, tumors exploit these regulatory pathways to escape T cell-mediated antitumor immunity. Tumor cells express ligands for immune checkpoint proteins such as the cytotoxic T lymphocyte-associated protein 4 (CTLA-4 also known as CD152), the programmed cell death 1 (PD-1 also known as CD278), and Lymphocyte Activation Gene-3 (LAG-3 also known as CD223) receptor molecules expressed on T lymphocytes. Tumor-expressed ligands activate these receptors, diminishing T-cell responses against the tumor. ICIs currently utilized in clinical practice are monoclonal antibodies that target these molecules: CTLA4, PD-1, PD-L1 (Programmed death ligand -1) and more recently LAG-3. These therapeutics block the receptor-ligand binding and release the inhibitory signaling, allowing T cells to continuously recognize and attack Tumor cells. The survival benefit of ICIs has been demonstrated in multiple randomized clinical trials, making them a mainstay therapy for various tumors. However, they are not without trade-offs. The remarkable efficacy of ICIs is limited by their potential autoimmune and inflammatory side effects known as immune-related adverse events (irAEs). IrAEs occur in about two-thirds of ICIs recipient requiring cessation of therapy in nearly 40 percent of patients (1). Autoimmune toxicities involve multiple organ systems such as the skin, gastrointestinal tract, liver, lungs, and endocrine system. Fortunately, most systemic, and organ-specific irAEs present with mild to moderate symptoms that can resolve spontaneously, with discontinuation of therapy or glucocorticoid therapy. In contrast to other organ-specific IrAEs, cardiotoxicities are rare, albeit with a high case fatality when they occur (1, 2). For example, the incidence of myocarditis in patient receiving ICI therapy ranges from 0.04% to 1.14%but with an associated mortality of 25% to 50% (3, 4). The potentially fatal outcome of cardiac irAEs warrant prompt intervention with supportive care and glucocorticoid therapy. Unfortunately, the rareness of this condition makes it difficult to obtain sufficient data and knowledge about these serious adverse events to form strategies for early detection, assessment, and management. As a result, the understanding of autoimmune cardiotoxicity remains limited, although rapidly evolving. In this paper, we provide an updated review of the literature on the pathologic mechanisms, diagnosis, and management of autoimmune cardiotoxicity as a result of ICIs and their combinations, and provide perspective on potential strategies and ongoing research developments to prevent and mitigate their occurrence.

## 2 Immune checkpoint inhibitors

There are at least nine US Food and Drug Administration-approved ICIs as of early 2022. These include an anti-CTLA4 monoclonal antibody (Ipilimumab); four PD-1 blocking monoclonal antibodies (Nivolumab, Pembrolizumab, Cemiplimab, and Dostarlimab); and three anti-PD-L1 antibodies (Atezolizumab, Durvalumab, and Avelumab) and one LAG-3 antibody (Relatlimab). **Table 1** shows clinical indications of each ICI approved by the FDA. Tremelimumab, an anti CTLA-4 monoclonal antibody has an orphan drug designation and is currently under investigation as a combination regimen with other ICIs (clinicaltrial.gov). In addition, some newer anti-PD-1 ICIs, such as Sintilimab, Tislelizumab, Toripalimab, and Camrelizumab, which the National Medical Product Administration of China has approved, are currently undergoing Phase II/III testing. Some emerging anti-PD-L1 currently under investigation include Cosibelimab, KN035, CA-170, BMS-986189, etc. (5) .

**Table 1:** Current FDA approved Immune Checkpoint Inhibitors and their indications.

Drug	Target	FDA Indication	FDA approval Year
**Ipilimumab**	CTLA-4	Melanoma, colorectal cancer, renal cell carcinoma	2011
**Nivolumab**	PD-1	Melanoma, Head and neck squamous cell carcinoma, Non-small cell lung cancer, Small cell lung cancer, Hodgkin’s lymphoma, Hepatocarcinoma, colorectal cancer	
**Pembrolizumab**	PD-1	Melanoma, non-small cell lung cancer, non-squamous cell lung cancer, renal cell carcinoma, classic Hodgkin's lymphoma, gastric or gastroesophageal junction adenocarcinoma, urothelial carcinoma, cervical cancer, large B-cell lymphoma, Merkel cell carcinoma	2014
**Cemiplimab**	PD-1	Cutaneous squamous cell carcinoma	2018
**Dostarlimab**	PD-1	Recurrent Endometrial cancer	2021
**Avelumab**	PD-L1	Merkel cell carcinoma, urothelial carcinoma, renal cell carcinoma	2015
**Atezolizumab**	PD-L1	Urothelial carcinoma, non-small cell lung cancer, breast cancer, non-squamous non-small cell lung cancer, small-cell lung cancer	2016
**Durvalumab**	PD-L1	Urothelial carcinoma, non-small cell lung cancer	2016
**Relatlimab**	LAG-3	Advance and metastatic melanoma	2022

All ICIs exert their antitumor activity by reversing the T cell tolerance towards tumor cells that is mediated by their checkpoint proteins. The mechanism of their toxicities, including cardiac toxicity, relates to this process. Thus, understanding T cell activation and their inhibition is needed to understand ICIs toxicities.

### 2.1 Modulators of T lymphocyte activation and tolerance

T lymphocytes serve as one of the prime mediators of the adaptive immune response against tumors. T cell immune checkpoint receptors are a wide variety of molecules found on T cells that are known to modulate the signaling pathways involved in the activation of antigen-specific, including anti-tumor responses (6, 7) . Activating T cell receptors include the T cell receptor complex and costimulatory molecules such as CD28, OX40, GITR (Glucocorticoid-induced TNF receptor family-related protein), CD137, CD27, HVEM (herpesvirus entry mediator). Inhibitory T-cell receptors that mitigate against T cell activity include but are not limited to CTLA-4, PD-1, LAG-3 (lymphocyte activation gene-3), TIM-3 (T cell immunoglobulin and mucin domain-containing protein 3), BTLA (B- and T-cell lymphocyte attenuator), and VISTA (V-domain Ig Suppressor of T-cell Activation) and the TIGIT (T cell immunoglobulin and ITIM domain). The capacity to develop an immune response is largely a consequence of the balance of stimulatory versus inhibitory signaling which can result in autoimmunity, as seen in cardiac pathologies following ICI treatment. There are other lesser understood intracellular metabolic pathways such as the indoleamine 2, 3-dioxygenase (IDO), and arginase in tumors and myeloid cells that also play a critical role in activating immune cells (8). More also, some other immune checkpoints are now known to play a critical role in the modulation of other subsets of immune cells aside of T cells (e.g., CD40 for B cells and TIGIT for NK cells) (9, 10). However, the current clinically utilized ICIs exploit the membrane-bound immune checkpoint proteins (CTLA-4, PD-1, PD-L1, and the more recent LAG-3). Cardiotoxicities from these immunotherapeutic are, therefore, our focus in this review.

### 2.2 Mechanism of immune checkpoint inhibition

T lymphocyte activation involves the following steps. First, antigen-presenting cells (APCs) process antigens to load antigenic peptides onto their major histocompatibility complex (MHC) molecules for recognition by a T cell that displays a cognate T cell receptor (TCR) and a co-stimulatory CD28 receptor for B7-1 (CD80)/B7-2(CD86) expressed by the APC (7). This primarily occurs either in lymphoid tissues for priming or peripheral tissues for secondary responses. In lymphoid tissue, T cells are activated when their TCRs bind to their cognate MHC-peptide complex presented by APCs in conjunction with concurrent CD28 binding to B7-1/B7-2. This initial response to antigen causes induction of CTLA-4 within the T cells, which is contained within intracellular vesicles of naive T cells and is then transported to the cell surface and expressed as a membrane molecule. The membrane-bound CTLA-4 signals to dampen and maintain a controlled level of T cell activation. T cells stimulated in peripheral tissues mainly express PD-1 rather than CTLA-4. Unlike CTLA-4, PD1 expression is upregulated transcriptionally at the mRNA level in response to inflammatory signals (such as IFN-γ) that are produced by activated T cells (7).

**Figure 1:**
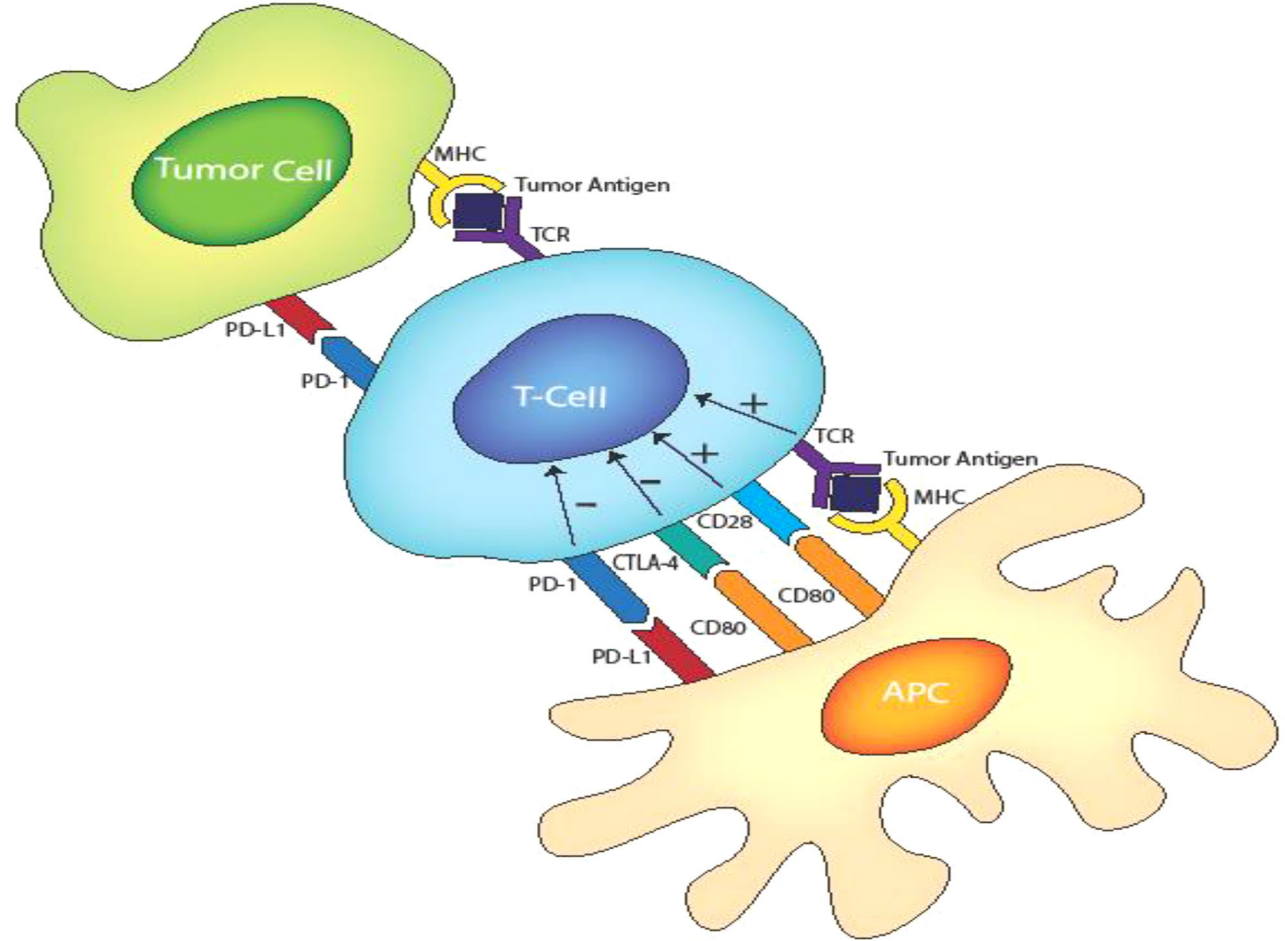
T cell activation and inhibitory receptors-ligand interactions involving TCR/MHC class II. CD28/D80. CTLA-4/CD80 and PD1/PD-L1.

CTLA-4 4 is a CD28 homolog with a stronger binding affinity for B7 that CD28. In the later phases of an immune response, membrane-bound CTLA-4 interacts with the B7 molecules on APCs, blocking their interaction with CD28 and thereby decreasing the T cell activation state which can render the cells anergic. Similarly, PD-1 binds to ligand PD-L1 and PD-L2 on the APCs to inhibit T-cell reactivity. Excessive induction of PD-1 on T cells in the setting of chronic inflammation and antigen exposure have been observed to cause T cell anergy. **Figure 1** shows the CD28/CD80, CTL4/CD80 and PD1/PDL1 inhibitory ligand interaction. LAG-3 inhibits activation of T cells in a similar fashion to CTLA-4 and PD-1. It is co-expressed with PD-1 in activated T cells, natural killer (NK) cells and APCs with its main ligand is the MHC class II, to which it binds in place of CD4 (a receptor of TCR) to dampen T cell activation (11, 12) . These dampening effects are needed in normal physiologic conditions to prevent T cell over-activity and maintain self-tolerance during a T cell response to invading pathogens and other antigen sources. However, tumors exploit these regulatory pathways by expressing these inhibitory ligands thereby interfering with the ability of T lymphocytes to direct anti-tumor immunity. These inhibitory processes can be reversed by ICIs to promote cancer immunotherapy. Anti CTLA-4, PD-1/L1 and LAG-3 antibodies restore the activity of anti-tumor T cells through blocking CTLA-4/B7, PD1/L2-L2, and LAG-3/MHC class II interactions respectively. However, the precise understanding of the immunostimulatory mechanisms of various ICIs remain under investigation. For example, recent pre-clinical studies implicating CTLA-4 as an intrinsic positive regulator of regulatory T cell (Treg) as opposed to merely a negative regulator of T effector cells are noteworthy (13) and LAG3 blockade have also been shown to interfere with the suppressive activity of Treg cells (11).

### 2.3 Clinical benefit of immune checkpoint inhibition

**Anti CTL4-A therapy:** Ipilimumab prolonged overall survival (OS) in patients with stage III or IV melanoma in a clinical trial, leading to its approval in 2011 (14). A combination therapy of Ipilimumab and Nivolumab, which targets PD-1, was subsequently approved for melanoma following data from the Checkmate 067 trial, which demonstrated an OS benefit for the combination therapy versus Ipilimumab monotherapy (15). It’s indication further expanded to include renal cell carcinoma after the Checkmate 214 trial showed significant improvement in OS and progression-free survival (PFS) (16, 17). In the Checkmate 227 and Checkmate 9LA, Nivolumab plus ipilimumab as first-line treatment improved OS compared to chemotherapy in non-small cell lung cancer (NSCLC) (18, 19). The newer anti-CTL4A-4 Tremelimumab was granted an orphan drug designation after showing modest clinical efficacy for treating malignant mesothelioma in a phase II trial. Tremelimumab, however, failed to meet clinical endpoints in the DETERMINE trial (20, 21). Tremelimumab is currently tested for other tumor types and in combination therapy (22).

**Anti PD-1 therapy:** Nivolumab was approved by the FDA in 2014 based on the CheckMate-037 trial, which demonstrated an improvement in overall response rate with Nivolumab against standard-of-care chemotherapy in patients with advanced and progressing unresectable/metastatic melanoma (23). Its clinical use in melanoma has expanded since 2014 based on the Checkmate 067 and Checkmate 238, which demonstrated OS and PFS benefits combined with Ipilimumab (15, 24). Similar efficacy has been demonstrated for other disease sites. These include Checkmate 17/57 and CheckMate-032 trial (NSCLC) (25, 26) Checkmate-214 (Renal cell carcinoma), (17, 27) Checkmate-205 (Hodgkin Lymphoma), (28) Checkmate 275 (Urothelial carcinoma), (29) Checkmate-040 (hepatocellular carcinoma) (30) and Checkmate-141 (head and neck tumors). (31) Pembrolizumab combination superiority over prior standard of care in the KEYNOTE-407 and KEYNOTE-042 trial (NSCLC) (32, 33) KEYNOTE 181 (Esophageal cell carcinoma) (34), KEYNOTE-158 (metastatic small cell lung cancer) (35), KEYNOTE-426 (Renal cell carcinoma) (36), KEYNOTE-224 (Hepatocellular carcinoma), KEYNOTE-017 (Merkel cell carcinoma) (37), KEYNOTE-170 (B-cell lymphoma) (38), KEYNOTE-158 (Cervical cancer) (35) (39), KEYNOTE-059 (Gastric and gastroesophageal junction cancer) (39), KEYNOTE-158(MSI-h dMMR cancers) (40), KEYNOTE-048 (Head and neck cancers) (41), KEYNOTE-087 (Hodgkin lymphomas) (42), KEYNOTE-006 (Melanoma) (14), and KEYNOTE-045 (Urothelial cancers) (43).

**Anti PD-L1 therapy:** Atezolizumab improve OS in the IMpower150 trial, as first-line treatment for metastatic NSCLC with no EGFR/ALK mutation when used in combination with standard chemotherapy than standard chemotherapy alone (44). Other trials with demonstrated superiority of PD-LI inhibitors and standard verse standard of care alone include the IMvigor210 trial for locally advance and metastatic urothelial cancers (45), Impassion-130 trial for triple negative breast cancer (46), IMpower133 for extensive stage small cell lung cancer (47). Avelumab demonstrated superiority in the JAVELIN trials (48) and Durvalumab in the PACIFIC trials (49).

**Anti-LAG-3 therapy:** Relatlimab in combination with Nivolumab showed an improved 12 months median progression free survival (47.7% vs 36%) in patients with previously untreated metastatic or unresectable melanoma when compared to Nivolumab monotherapy in the RELATIVITY-07 trail (50).

## 3 Cardiac irAEs of immune checkpoint inhibitors

Cardiac IrAEs have been reported in association with anti CTLA-4, anti PD-1, and their combinations. Reported cardiac toxicity is diverse, involving various cardiac tissues.

### 3.1 Epidemiology

The exact incidence of cardiac IrAEs resulting from ICI therapy have been difficult to quantify as early clinical trials testing efficacy of ICIs did not routinely evaluate for changes in cardiac function and myocardial injuries. Limited epidemiological data can be obtained from manufacturer safety databases, the World Health Organization (WHO) pharmacovigilance repository, (3) the FDA Adverse Event Reporting System (FAERS) database, and retrospective studies including meta-analysis of existing data and case reports. However, estimates from each source vary significantly. There is a possible underestimation of the incidence of cardiac irAEs for a host of reasons, ranging from the vagueness in its clinical presentation, the potential overlap with other cardiovascular disease and comorbidities, and a poor awareness of this condition (51). The WHO database reported higher incidence of ICI irAEs likely due to increased use of ICIs and improved recognition of their toxicities. Data from WHO database suggests myocarditis and arrhythmias as the most common cardiac irAEs. **Table 2** shows selected cardiac morbidities as a percentage of overall cardiac irAEs reported on Vigibase for each ICI as of 2022. In a 2020 systematic review and meta-analysis, 0.1%-0.9% for myocarditis, 0.1%-1.0% for pericardial effusion, 0.0%-0.5% for cardiac failure, 0.3% for cardiomyopathy, 4.6% for atrial fibrillation, 0.0%-0.7% for myocardial infarction, and 0.1%-0.8% for cardiac arrest (52). Pharmacovigilance reporting systems may be limited by under-reporting, reporting bias, and a lack of information on population exposed to the drug. The risk associated with a drug is therefore difficult to quantify accurately in these databases (53–55).

**Table 2:** Select cardiac pathology as a percentage of overall cardiac irAEs reported on Vigibase for each ICIs as of 2022*(VigiAccess, July 2022)*.

	Anti C TL4-A	Anti PD-1		Anti PD-L1					Anti-LAG- 3
**Select Cardiac irAEs**	Ipilimu mab	Nivolu mab	Pembrolizu mab	Atezolizu mab	Aveluma b	Cemipli mab	Dostarlim ab	Durvalum ab	Relatlimab
**Reporting timeline**	2009-2002	2014-2022	2015-2022	2015-2022	2015-2022	2018-2022	2019-2022	2014-2022	2017-2022
**Cardiac irAEs as percent of all irAEs (%)**	2	2	2	2	3	3	5	3	6
**Myocarditis (%)**	22.7	20.7	19.9	15.8	20	27.3	14.2	21.4	33.3
**Atrial Fibrillation (%)**	16.3	11.2	9.7	15.4	14.4	7.6	7.1	11.6	n/a
**Myocardial Infarction (%)**	6.1	6.5	6.2	7.9	2.3	6.1	7.1	5.8	11.1
**Cardiac arrest (%)**	6.6	5.0	5.0	5.8	10.0	3.0	21.4	7.1	n.a
**Cardiac Failure (%)**	5.6	8.3	8.3	9.1	10.0	16.7	0.0	5.4	11.1

Vigibase do not provide data on fatality. Wang and colleagues in a 2018 meta-analysis of 112 trials involving 19,217 patients showed toxicity-related fatality rates of 0.36% for anti-PD-1, 0.38% for anti-PD-L1, 1.08% for anti-CTLA-4), and 1.23% for combined anti-PD-1/PD-L1 plus CTLA-4 therapy (2). A 6 year (2011-2017) analysis of the Danish registry demonstrated an absolute risks for cardiac irAEs of (6.6–9.7%) with anti-PD1 and anti-CTL4 therapy, significantly higher than reports from pharmacovigilance studies (56). However, this study only included patients with malignant melanoma and lung malignancies which are generally considered high risk malignancies for irAEs. Moreover, the determination of what entails a cardiac irAE, which is not consistent between reports, may explain some discrepancy between various data repositories (57). Evidently, mortality is more frequent with combination PD-1/CTLA-4 blockade (58). There are currently no mortality data for anti-LAG-3 therapy and Vigibase cardiotoxicity data on Relatlimab should be characterized with caution due to a low sample of only 66 adverse events. Additional large prospective studies are needed to provide more precise estimates of the actual incidence and fatality rates of cardiotoxicity arising from ICI immunotherapy.

## 4 Mechanism of ICI induced cardiac IrAEs

The exact mechanism of ICI-associated cardiotoxicity is not yet fully understood (59). Proposed mechanisms include: (i) Direct destruction of cardiac tissue by deregulated, activated autoimmune T lymphocytes; (ii) Indirect destruction of cardiac structures by pro-inflammatory cytokines and other molecules released by ICI deregulated T lymphocytes and the cells that they activate, such as macrophages; (iii) Recognition of cardiac self-antigens by autoantibodies to promote cell-mediated cardiotoxicity. These mechanisms can involve single or multiple cardiac structures resulting in pathologies.

### 4.1 Direct cellular destruction of cardiac tissue

**Figure 2:**
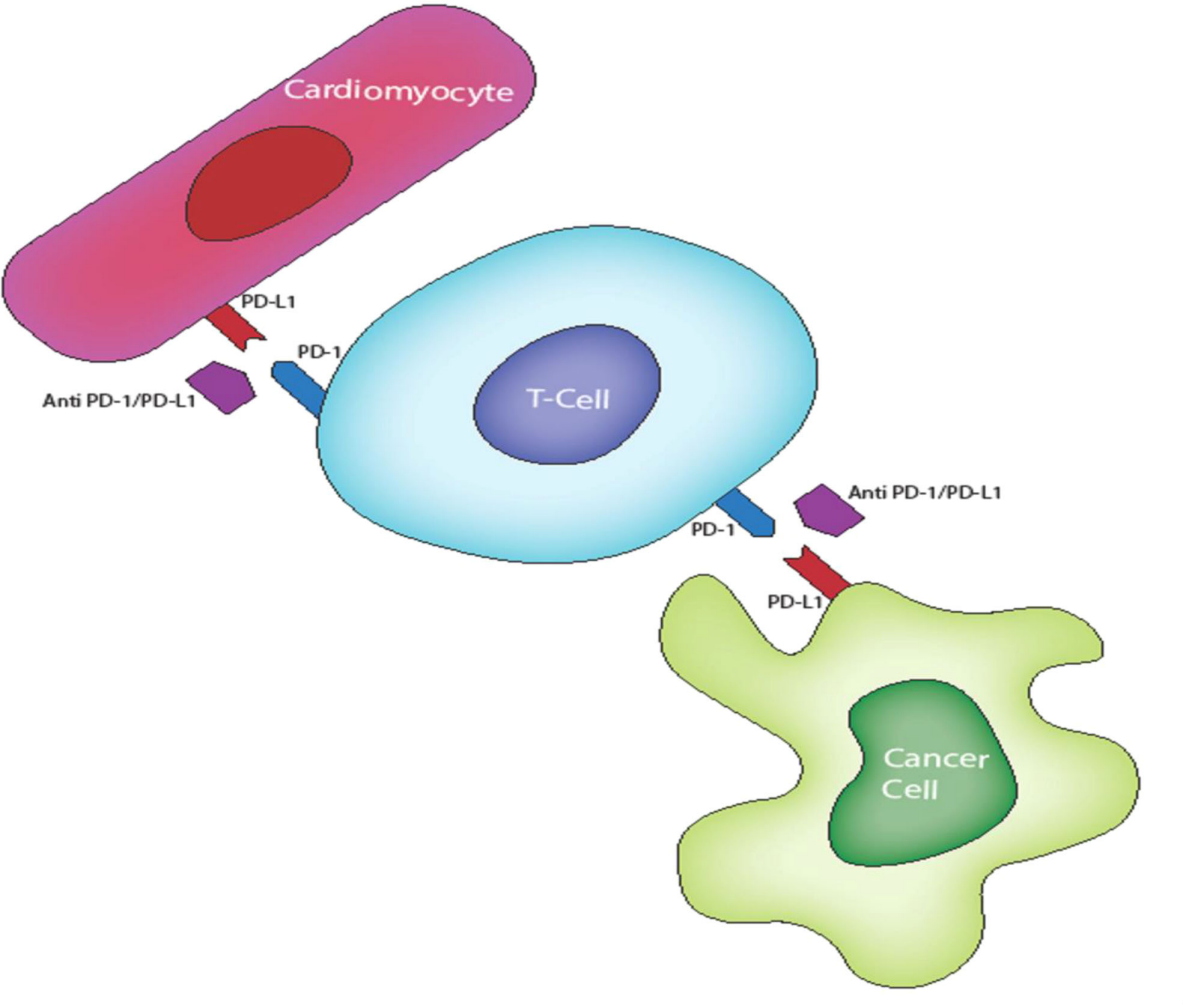
PO-L1 expression on cardiac tissues confers protection from activated T cell *via* P0-1/PO-L1 inhibition of T cells. This inhibition is lost in ICI therapy resulting in an autoimmune T lymphocyte destruction of cardiac tissues.

Cardiac cells, like APCs and certain cancer cells, are now known to activate CTLA-4 and PD-1/PD-L1 pathways to maintain self-tolerance of cardiac structures during T lymphocyte responses to stress and stimulatory antigens under physiological conditions (60) (see **Figure 2**). CTLA-4 and PD-/PD-L1 blockade likely interrupt this immunologic homeostasis thereby causing auto-immune cardiac toxicity mediated by deregulated T-lymphocytes. Evidence for this theory stem from histological and immunohistochemical analyses demonstrating membrane and cytoplasmic expression of PD-L1 in injured cardiac tissue (61, 62). PD-L1 expression is higher in cardiac tissue samples from patients with ICI-associated myocarditis, which is consistent with lymphocytic myocarditis as histologically characterized by myocardial infiltration of macrophages and CD4+/CD8+ T lymphocytes (63, 64). In a preclinical study, Grabie et al. demonstrated the expression of PD-LI on cardiac endothelium which has a cardio-protective effect against T lymphocyte-mediated cardiac injury (65). Preclinical insights from genetic and manipulation of immune checkpoint pathway have further bolstered this theory. For example, PD-1 and CTLA-4 knockout mice develop rapid lymphoproliferation and fatal T cell mediated myocarditis (66).

Cellular infiltration of cardiac myocytes in irAEs may also be due to the immune polarization effects of ICIs (67) . For example, anti-PD1 has been found to transduce immunoregulatory signals that modulate macrophage polarization to pro-inflammatory phenotype *via* the inhibitory effects of microRNA-34a (miR-34a) on the Krüppel-like factor 4 (KLF4) signaling pathway. Consistent with this finding, among other activities, the transcription factor KLF4 has anti-inflammatory properties with a cardiac protective effect. Xia and colleagues hypothesize that miR-34a mediated inhibition of the KLF4 pathway leading to inflammatory macrophage activity may account for the cellular infiltration and destruction of cardiac tissues seen in ICI therapy. In their *in-vivo* experiment, anti-PD1 treatment was shown to induce polarization of pro-inflammatory macrophages accompanied by increased MicroRNA-34a expression and decreased expression of KLF4, resulting in cardiac injury 67).

### 4.2 Cardiac antigen immune reactivity

There is ample of evidence to suggest the existence of common T-cell receptors or epitopes between certain cardiac myocytes and tumor (68, 69). This shared antigen theory is supported by the relatively early onset of myocarditis observed after initiating ICI therapy in a select group of patients. It is quite possible that a pre-existing molecular mimicry that allows an immune evasion for these cardiac cells in a similar fashion to the tumors become disrupted, predisposing these patients to the development of myocarditis when treated with ICIs (69). However, multiple questions remain to be answered with respect to this hypothesis such as the nature of these epitopes, how they elicit an immune response, and how immune effectors are targeted to cardiac tissue. While these questions abound, recent translational studies suggest a second hit may be necessary to initiate cardiac immune reactivity (70). In a study by Michel and colleagues, mice models with transplanted tumors developed left ventricular (LV) dysfunction with the initiation of ICI therapy. In contrast, LV dysfunction was undetectable in tumor-free mice receiving the same ICI therapy. This finding has led to the postulation of a second hit theory, which argues that a form of systemic stress induced by the presence of the tumor may be required to initiate the cardiac immune reactivity in predisposed patient (70). In addition, anti- PD-1 therapy is now recognized to drive the development of auto-antibodies against cardiac specific proteins. Okazaki et al. demonstrated that mice deficient in PD-1 develop autoimmune dilated cardiomyopathy with production of high-titer autoantibodies against the cardiac-specific protein cardiac troponin I (cTnI) (71, 72). Further investigation demonstrates that the anti cTnl autoantibodies induces heart dysfunction and dilation through chronic stimulation of Ca^2+^ influx into cardiomyocytes (71, 72). Other auto-antibodies induced by ICI therapy with the potential to initiate or escalate cardiac irAEs include antibodies reactive with acetylcholine receptors, striated muscle cells, mitochondria, alanyl-tRNA synthetase, signal recognition particle (SRP), and 3-hydroxy-3-methylglutaryl-coenzyme A reductase (73–76). These auto-antibodies have been associated with myocarditis, primarily mediated through cross reactivity with cardiac striated muscle antigens and/or inducing antibody-dependent cellular cytotoxicity (ADCC) (73–76).

### 4.3 ICI induced cytokines release

**Figure 3:**
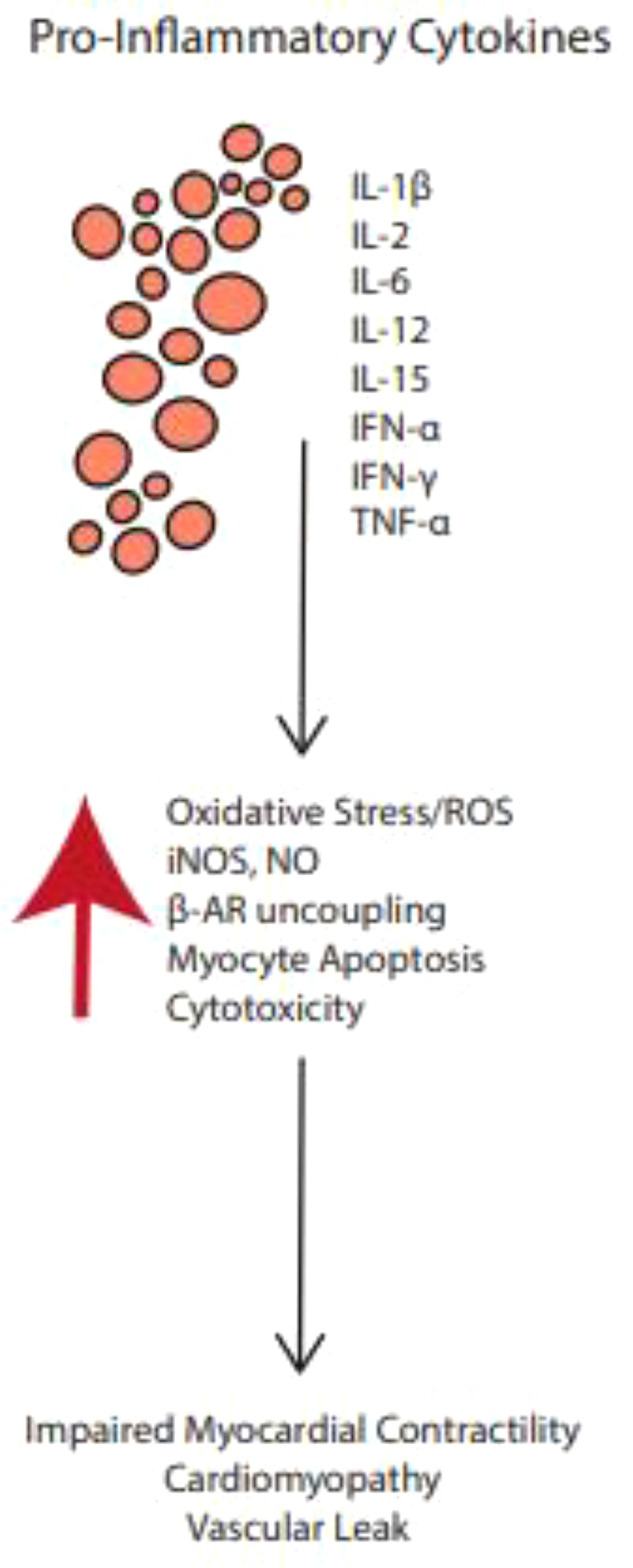
Pro-inflammatory cytokines upregulated by ICI therapies may activate certain T cell subsets, leading to a constellation of non-specific inflammatory processes known as the cytokine release syndrome.

The production of pro-inflammatory cytokines is upregulated by therapies that activate certain T cell subsets, leading to a constellation of non-specific inflammatory processes known as the cytokine release syndrome (CRS) (77, 78) (see **Figure 3**). In CRS, T cells, NK cells, APCs and endothelial cells, release a variety of cytokines at supraphysiologic levels (77) Interleukin-6 (IL-6) is most implicated in CRS (77). Other molecules associated with CRS include interferon-gamma (IFN-γ), tumor necrosis factor-alpha (TNFα); nitric oxide (NO); nitric oxide synthase (NOS); and reactive oxygen species (ROS) ^18^. These cytokines and radicals can have cytotoxic effects on cardiac myocytes, resulting in arrhythmias, conduction abnormalities, impaired contractility, and other cardiac anomalies (78, 79). CRS is however less common with ICIs when compared with other novel cancer immunotherapeutic such as the chimeric antigen receptor (CAR) T cell therapy (80). Findings from Vigibase data on adverse drug reactions suggests CRS incidence to range from 0.05% to 0.14% for ICIs, and more common with anti-PD1/PD-L1 combination therapies (77).

### 4.4 Dysregulation of myocardial metabolism

**Figure 4:**
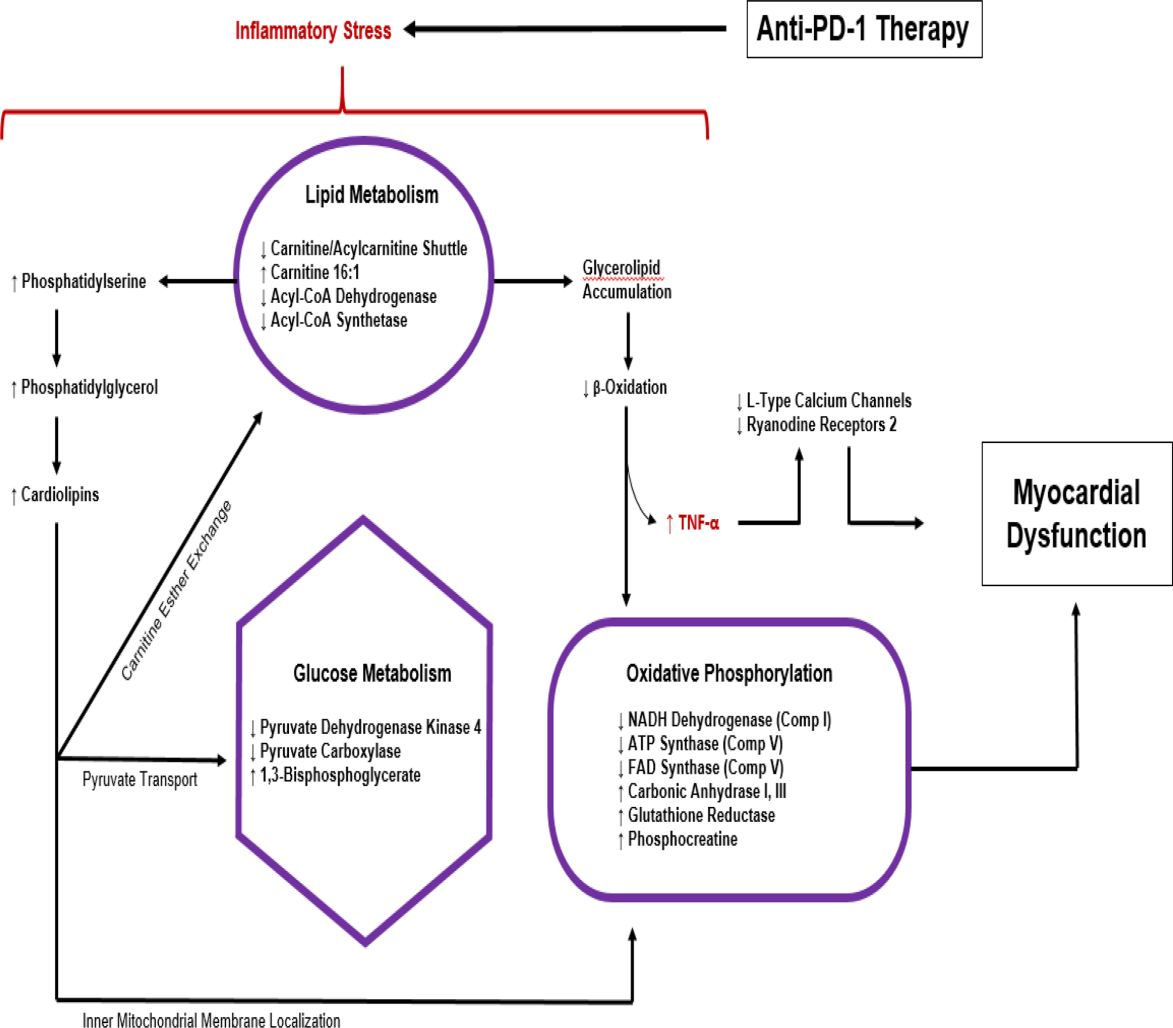
Metabolic response to anti-PD1 therapy based on substrate analysis in experimental model (70, 81).

Michel and colleagues propose a metabolic pathway leading to myocardial dysfunction due to anti-PD1 therapy based on substrate analysis in experimental model (see **Figure 4**) (70, 81) . Molecular analysis of cardiomyocytes from mice treated with anti-PD1 therapy shows metabolic disturbances including a reduction in metabolites such as carnitine/acylcarnitine carrier protein, acyl-CoA dehydrogenase, acyl-CoA synthetase pyruvate dehydrogenase kinase 4 (PDK-4), and pyruvate carboxylase with a concomitant increase in beta-oxidation substrates, cardiac TNF-alpha and 1,3-bisphosphoglyceric acid (70). These measures indicate changes in lipid and glucose metabolism capable of altering oxidative phosphorylation, mitochondrial function, plasma membrane permeability, and other cellular functions, ultimately leading to cell death. This dysregulation of myocardial metabolism seen with ICI therapy is likely to be a downstream effect of the immune/inflammatory pathologies caused by the cardiac irAE mechanisms already discussed above but may also drive currently underappreciated aspects of the disease process.

## 5 Clinical risk factors for ICI induced cardiac IrAEs

Identification of patients at risk for ICI induced IrAEs is difficult and an ongoing area of research. A risk predictive model is needed to provide a basis for the clinical use of ICIs, as well as a guide for the prompt management of ICI toxicities. Identified patient-related risk factors for cardiac IrAEs include pre-existing cardiovascular diseases, co-morbidities (such as hypertension and diabetes mellitus), age, sex, underlying autoimmune diseases, opportunistic pathogens, medications, tumor-related factors, and genetic predisposition. Therapy-related risk factors include the use of combinatorial cancer therapy (such as irradiation, chemotherapy, targeted therapies, and other ICIs or immunotherapies), specific ICIs and their dosage.

### 5.1 Patient related risk factors

Several possible baseline risk factors proposed for IrAEs in general have little prospective evidence to support their association with the development of cardiac specific IrAEs. Females have been reported to be associated with higher rates of IrAEs although this phenomenon lacks mechanistic explanations (82). Age group and BMI as a risk factor have yielded conflicting reports in retrospective studies (82). One retrospective study demonstrated an association of ICI IrAEs with patient BMI. IrAEs were found to increase by 9% with every BMI increase by 1 kg/m^2^ (83). The occurrence of certain toxicities varies depending on the type of malignancy and/or pathway blocked. Patients with lung cancer are notable for increased odds of irAEs or irAEs requiring hospital admission when compared to patients with other malignancies (melanoma OR (odd ratio): 0.70, renal cell carcinoma OR: 0.71, other malignancy OR: 0.50) (84). Hazard ratios of 2.14 (95% CI 1.50-3.05) in patients with lung cancer and 4.30 (1.38-13.42) and 4.93 (2.45-9.94) have been demonstrated in patients with malignant melanoma treated with anti-PD1 and anti-CTLA-4, respectively (56). Furthermore, a circulating neutrophil/lymphocyte ratio greater than 3.0 at the time of starting treatment has been correlated with a lower risk of IrAEs (83). Pre-existing auto-immune disorders may also increase risk for ICI IrAEs as reported in multiple case series (85). However, this remains unclear. Baseline cardiac pathologies is also a risk factor. In the Phase III Javelin Renal 101 trial of ICI and targeted therapy combination, patients with elevated baseline troponin suggestive of baseline cardiac pathologies and autoimmune diseases were shown to have higher risk of major cardiac irAEs when compared to patients with low baseline troponin values (86).

### 5.2 Therapy-related risk factors

#### 5.2.1 Combinatorial therapy

ICI combinations, either with other ICIs or with other oncologic therapies such as chemotherapy, radiation therapy, and targeted therapy have significantly improved prognosis for many cancers. The cardiac irAEs of combination regimens involving ICIs and other conventional therapies is an active area of investigation as toxicities inherent to individual therapies may amplify with various combinations.

- **Dual ICI therapy:** Clinical benefits of combination ICI have been demonstrated in multiple randomized clinical trials. However, this often comes at a cost of exacerbated treatment toxicities. In a recent database review of over 14,000 patients who received ICI in the United Sates, combination ICIs (anti-PD-1/PD-L1 and CTLA-4) were associated with a more than two fold increase in odds of developing IrAEs requiring hospital admission which were particularly noticeable in lung malignancies (84). In this study, incidence of irAEs warranting hospital admission was 3.3% for patients treated with anti-PD-1 antibodies, 1.1% for patients treated with anti-PD-L1 antibodies, 3.9% for patients receiving anti-CTLA-4 antibodies, and 3.5% overall for all ICI antibodies as monotherapy. However, hospitalization rates was increased to 7.3% for patients on combination therapy (84, 87). Hu in his systematic review and meta-analysis of 2,551 studies with 20,244 patients reported an increased risk of cardiac arrhythmia with ICI combination (anti PD-1 and anti CTLA-4) therapy compared to either agent as monotherapy (OR 3.90, 95% CI: 1.08–14.06, p = 0.603) (88). Also, WHO database reports mortality from ICI-associated myocarditis to have an almost two fold increase with combination ICIs (60% versus 36%) when compared to patients who receive anti-PD-(L)1 monotherapy (58).
- **Chemotherapy and Targeted therapies:** Many conventional chemotherapies unfortunately have cardiotoxicities effects that can be amplified with ICIs whether delivered concurrently or sequentially. The hypothesized mechanisms of chemotherapy-induced cardiotoxicity vary by agents. For example, anthracyclines may have direct cellular toxicity *via* mitochondrial damage, with cumulative myocardial injury, resulting in both diastolic and systolic dysfunction (89). Taxanes cause myocardial damage *via* their effects on subcellular organelles, or through the induction of massive histamine release, and are associated with conduction disturbances and arrhythmias (89). 5-Fluorouracil has direct toxic effects on the vascular endothelium which can cause spasm of coronary vessels, platelet aggregation, and thromboxane formation, increasing thrombogenesis and cardiac injuries. The potentiation of these chemotherapy-associated and/or ICI-associated cardiac IrAEs in chemotherapy-ICI combination therapy is an active area of research. Meta-analysis however, demonstrates an increase in cardiac IrAEs when a chemotherapeutic agent is combined with ICI ^85^. In Hu’s study, PD-1 blockade plus chemotherapy exhibited a significant increase in all grades of myocardial disease when compared with chemotherapy alone (88). Aside from the conventional systemic therapies, cardiac toxicity in targeted therapies is increasingly also being recognized. Trastuzumab (an anti-erbB2) for example is known to cause left ventricular dysfunction and the induction of congestive heart failure. BRAF and MEK inhibitors can also cause a decline in left ventricular ejection fraction (90). There is a demonstrable risk of myocardial infarction, atrial fibrillation, and QTc prolongation with BRAF inhibitor therapy which is theorized to be caused by a BRAF-mediated alteration of the myocardial repolarization process (91). The potentiation of cardiac toxicities due to treatment with ICIs in combination with these targeted therapeutics has yet to be explored in a clinical trial. However, insights from pre-clinical studies demonstrate a 3 fold increased calcium overload and reduced viability of human cardiomyocytes treated with the combination of Pembrolizumab and Trastuzumab compared to cells treated with either reagent alone (92, 93). The pembrolizumab-trastuzumab combination, when compared to monotherapy, was also noted to increase inflammation affecting cardiac cells and cardiac fibrosis by enhancing the expression of NF-kB and interleukins (93).
- **Radiation Therapy**: The effects of radiotherapy (RT) on both tumors and its microenvironment involves a complex manipulation of immune system. Radiotherapy has potential to alter the tumor immune microenvironment to augment the antitumor effects of ICIs, specifically by releasing cytokines, endogenous danger signals, increasing the presentation of tumor-associated antigens by APC, and stimulating diversification of the anti-tumor T cell repertoire (94). Wang and colleagues demonstrated RT and anti-PD1 synergy to improve clinical endpoints may result from RT overcoming PD-1 inhibitor resistance by inducing the production of type I interferon (IFN) leading to an enhancement of MHC class 1 expression (95, 95). Lee and colleague showed radiation therapy at an ablative dose can have an anti-tumor effects that are dependent on cytotoxic T-cells (96). Other studies have observed an abscopal effect where radiation therapy of primary tumor could have a potent effect on non-irradiated tumor cells (94) (94). However, combined radiotherapy and ICI may also affect both the type and severity of immune related toxicities, including cardiotoxicity. For example, the combination of thoracic radiation and PD-1 blockade can exacerbate radiation-initiated cardiac inflammation and cardiotoxicity (97, 98).

#### 5.2.1 Dosage of ICI

The safety of ICIs given in combination with a variety of other cancer agents is clearly dependent on the dosage administered (99) . There is also evidence that this is the case for the risk of cardiac IrAEs (100). However, establishing safe doses for novel combination therapies involving ICIs has been challenging in face of the limited clinical experience with their utilization (99). In a meta-analysis by Bertrand and colleagues, the risk of developing all irAEs was dependent on dosage, with their incidence evaluated as 61% (95% CI, 56-66%) for ipilimumab at a dose of 3 mg/kg and 79% (95% CI, 69-89%) for ipilimumab at a dose 10 mg/kg (101). Another meta-analysis of 2,551 studies including 25 clinical trials and 20,244 patients treated for advanced melanoma show a decreased risk for all severe IrAEs with ipilimumab at 3 mg/kg every 3 weeks; pembrolizumab at 10 mg/kg every 2-3 weeks; and Nivolumab at 3 mg/kg every 2 weeks when compared with ipilimumab at 10 mg/kg every 3 weeks (102). The irAEs were unspecified in this study (102). Hu’s cardiac specific meta-analysis however did not show any significant difference in cardiac IrAEs between ipilimumab at a dose of (3 mg/kg q3w) versus (10 mg/kg q3w) (88). Nivolumab at 3 mg/kg plus ipilimumab at 1 mg/kg also showed no increased risks of coronary artery disease compared with a dose of ipilimumab at 3 mg/kg plus Nivolumab at 1 mg/kg. Similarly, compared with a dose of 10 mg/kg q2w, a dose of 10mg/kg q3w PD-1 inhibitor (pembrolizumab) did not show significantly increase risks of cardiac failure (88). Dosage for a combination including ICI and a different kind of immunotherapeutic (such as CART-T) or a biologic agent is much more complex and requires additional study at this time (99).

## 6 Clinical diagnosis and management

### 6.1 Clinical manifestation

One main prerequisite for managing cardiac IrAEs is the knowledge and awareness of this complication. Subtle signs and symptoms which may become progressive need to be adequately interpreted to initiate management and avert complications. Manifestations of cardiac irAEs range from a subclinical rise in cardiac biomarker and vague symptoms such as malaise to overt symptoms of chest pain, dyspnea, palpitations, progressive fatigue, pre-syncope and syncope that can lead to multiorgan failure, cardiogenic shock, and cardiac arrest. (59, 103). These symptoms may be obscured by other non-cardiac irAEs such as myositis, hypothyroidism, pneumonitis, or other symptoms related to the primary malignancy or comorbid conditions. The median time to onset of clinical manifestation of cardiac irAEs is 6 weeks (typically 3 to 9 weeks) but can range from 2 to 54 weeks (104), typically corresponding to the period after the first and third infusion (105). The average time until symptoms vary for each ICI type, cancer type, type of cardiotoxicity, and delivery with other therapeutics (104). On literature review, the anti-PD-L1 ICIs were found to have an earlier median time to presentation of symptoms (1-9 weeks for Atezolizumab and Durvalumab) (104). The anti-CTLA-A agent ipilimumab had a longer median onset time of 10 weeks, however in combination with nivolumab this median time was reduced to 6 weeks (104).

### 6.2 Clinical investigation

A detailed history, review of systems, and physical exams is required to exclude other cardiac diseases. Blood tests, electrocardiograms (ECGs), chest X-ray, and trans-thoracic echocardiograms (TTEs) are needed for diagnosis and management. Laboratory tests typically include the assessment of serum levels of cardiac troponins (including cardiac troponin I [cTnI] and troponin T [cTnT]), creatine phosphokinase (CPK), creatine kinase (CK), and creatine kinase-myocardial band (CK-MB). Others include brain natriuretic peptide (BNP), and N-terminal pro-brain natriuretic peptide (NT-proBNP) (59, 103). Additional testing such as stress tests, cardiac catheterization, and cardiac MRI may be guided by the cardiologist (103).

#### 6.2.1 Laboratory investigations

A hallmark of ICI induced myocarditis is an increase in serum cardiac biomarkers, notably troponin, BNP, NT-proBNP, and CK-MB which are further discussed in the biomarker subsection of this review.

#### 6.2.2 Electrocardiography

ECG is often a first-line test to identify patients with suspected cardiac irAEs. A 12-lead ECG should immediately be performed once a patient complains of chest pain, palpitations, dizziness, dyspnea, or any other concerning cardiac symptom (106). Abnormal ECGs have been reported in 40–89% of patients with ICI related toxicities. ECG abnormalities that may raise suspicion of cardiotoxicity include abnormal PR interval, ST-segment depression and elevation, atrioventricular block, ventricular arrhythmias, T-wave inversions, and new Q waves anomalies (107), (103, 106). T wave changes are the most common ECG abnormalities seen ECG changes in ICI cardiac events (107), (103, 106). ECG should be carefully interpreted with context to the patient as anomalies are common in the cancer patient population which do not always indicate a cardiac irAEs. Collecting a baseline ECG allows for recognition of any change occurring during ICI therapy, facilitating early diagnosis of associated cardiotoxicity (106).

#### 6.2.3 Cardiac imaging

For concerns of an acute coronary syndrome, emergency coronary angiography may be indicated for patients presenting with abnormal cardiac biomarkers and ECG or ischemic symptoms. In addition, TTE could provide further insight into motion anomalies of the myocardium and left ventricular ejection fraction (LVEF) compromise. TTE anomalies may be detected at later stage of ICI-associated myocarditis. Cardiac MRI (CMR) has a diagnostic superiority to TTE because it can identify fibrosis and inflammation tissue characteristics in the early course of the disease. ICI myocarditis is typically defined using the modified Lake Louise Criteria (108, 109). An analysis of clinical, CMR, and histopathological findings of patients on ICIs from international registries and retrospective studies shows that T1 mapping and application of the modified Lake Louise I or the updated Lake Louise II criteria provides important diagnostic value and prognostic value in patients with ICI-associated myocarditis (108, 110, 111). CMR and echocardiographic findings of impaired global circumferential strain, global radial strain, and global longitudinal strain in patients with an ICI associated myocarditis have been reported by many studies (112, 113). Other studies also showed a low sensitivity of CMR in detecting cardiac irAEs with features such as septal late gadolinium enhancement (LGE) seen only in 48% of patients (114). More also, LGEs result from the changes contrast uptake and washout patterns within the extracellular space could be seen in most myocardial injuries and therefore not specific for ICI-associated myocarditis (115). Further studies are needed to characterize cardiac MRI criteria for ICI-associated toxicities.

#### 6.2.4 Endomyocardial biopsy

Endomyocardial biopsy (EMB) which is gold standard for the diagnosis of ICI myocarditis, should be considered for patients with concerns for myocarditis based on cardiac imaging, cardiovascularly unstable patients, and patients who fail to respond to initial treatment with steroids. EMB could also aid definitive diagnosis when diagnosis is in doubt. Myocardial features identified on EMB for ICI-associated myocarditis include interstitial fibrosis, lymphocyte infiltration, T cells (CD4^+^, CD8^+^), macrophage infiltration, and other inflammatory changes (116). Palaskas and colleagues recently developed a grading system for ICI myocarditis and myocardial inflammation by pathology findings on EMB and noted a correlation with clinical outcomes (see **Table 3A**) (117). Interestingly, the Palaskas et al. study identified patients with EMB confirmed grade 1 ICI induced myocarditis as a low-risk group that may be capable of continuing ICI therapy without immunomodulation (117). This finding is however difficult to routinely introduce to clinical practice give the need for an EMB for grading ICI-related myocarditis. Champion and Stone used EMB to classify ICI-associated myocarditis based on inflammatory cell accumulation in cardiac tissues into high-grade (>50 CD3+ cells/high power field) and low-grade (≤50 CD3+ cells/high power field) groups by EMB finding (118) (see **Table 3B**). High-grade patients had a fulminant clinical disease course leading to a hundred percent fatality, while patients with low-grade cell accumulation had a more indolent clinical course with a hundred percent overall survival (118). These findings illustrate the value of EMB assessment of the extent of inflammatory changes in cardiac tissue following ICI but standardized criteria are yet to be adopted for the histopathologic grading of ICI myocarditis (119) .

Table 3Current Pathology Grading criteria for ICI induced myocardial inflammation.

**Table:** 

3a. Palaskas et.al Grading Criteria (117)	
Grade	Pathologic features
**0**	Negative
**1- Myocardial inflammation**	Multifocal inflammatory infiltrates without overt cardiomyocytes loss by light microscopy
**1A**	Mild inflammatory cell score by immunohistochemistry (10-20 inflammatory cells/ high power field)
**1B**	At least moderate inflammatory cell score by immunohistochemistry (>20 inflammatory cells/ high power field)
**2- Definite myocarditis**	Multifocal inflammatory cell infiltrates (>40 inflammatory cells/ high power field)

**Table:** 

3b. Champion and Stone Grading Criteria (118)	
Grade	Immunohistochemistry
**Low Grade**	(50 CD3+ cells/high power field
**High Grade**	>50 CD3+ cells/high power field

### 6.3 Treatment of ICI-induced cardiac irAEs

Treatment of ICI cardiac irAEs requires collaboration between the oncologist and cardiologist. In all cases, empirical treatment for ICI cardiotoxicity should be started once the suspicion is high, even before confirmatory pathologic testing is obtained. The 2021 American Society of Clinical Oncology Clinical (ASCO) practice guidelines recommends holding ICI therapy starting with Grade 1 cardiac irAEs and a permanent discontinuation of therapy for Grade 2 or higher toxicities (see **Table 4**) (103). ASCO guidelines also recommend that all-grade toxicities have early administration of high-dose corticosteroids, typically 1-2 mg/kg of prednisone oral or intravenous depending on symptoms (103).

**Table 4:** ASCO grading for ICI induced myocarditis is based on biomarkers, ECG, imaging and clinical presentation (120).

	Grade 1	Grade 2	Grade 3	Grade 4
**Clinical**	Asymptomatic	Mild	Moderate	Moderate to severe
**symptoms**			(Symptom with	decompensation, IV
			mild activity)	medication or
				intervention required,
				life-threatening
				conditions
**Cardiac**	Abnormal	Abnormal	Abnormal	Abnormal
**Biomarkers**				
**ECG**	Abnormal	Abnormal	Abnormal	Abnormal
**TTE**	–	–	LVEF <50% or	LVEF <50% or
			regional wall	regional wall motion
			motion	
**Cardiac MRI**	–	–	Cardiac MRI	Cardiac MRI
			diagnostic or	diagnostic or
			suggestive of	suggestive of
			myocarditis	myocarditis

An immediate transfer to a coronary care unit is recommended for patients with elevated troponin or conduction abnormalities (103, 120). Patients with no immediate response to low dose steroid (1-2mg/kg) may receive high dose steroid (1 g daily intravenous methylprednisolone) with addition of other immunosuppressive therapy such as mycophenolate, infliximab, or anti-thymocyte globulin (103). ASCO clinical practice guidelines recommend a steroid taper of at least 4 to 6 weeks. Aggressive initial steroid strategy is also an option (500-1000 mg daily), especially in clinically unstable patients (4, 121). Mahmood et al retrospectively compared high dose versus low‐dose glucocorticoids and reports lower adverse events in patients who received high‐dose steroids (4, 121). Although selection criteria for high‐dose versus low‐dose steroids were unclear in this retrospective series, the authors recommend pulse dose steroids at 1000 mg daily, followed by 1 mg/kg daily of either oral or intravenous steroids (4, 121).

In steroid refractory cases, alemtuzumab, infliximab, tocilizumab, or rituximab and the CTLA4 agonist (abatacept) can be considered. Caution is needed with use of infliximab as it has been associated with heart failure and is contraindicated at high doses in patients with moderate to severe heart failure. Plasmapheresis has also been used, with the goal of accelerating removal of the contributing drug (as well as any potential circulating autoantibodies). This approach is important with ICIs because their half-lives are extremely long: 14.5 days for Ipilimumab, 25.0 days for pembrolizumab, 26 to 27 days for Nivolumab and 27.0 days for Atezolizumab. Supportive management can entail inotropic therapy and even mechanical circulatory support, including extracorporeal membrane oxygenation, as a bridge to recovery, as has been shown in patients who developed fulminant myocarditis with cyclophosphamide and ICIs. Current treatment recommendations are notably based on anecdotal evidence and the life-threatening nature of cardiac complications.

## 7 Biomarkers

Molecular biomarkers are needed to predict which patients will experience cardiac IrAEs from ICI therapy. Several biomarkers such as the expression of programmed cell death ligand 1 (PD-L1), tumor mutation burden (TMB), and microsatellite instability-high (MSI-H)/mismatch repair-deficiency (dMMR) have proven to be predictors for anti-tumor efficacy of ICIs (122, 123). However there remains a pressing clinical need for the identification of biomarkers that can predict toxicities as well as help filter out the patients who may benefit most from these costly therapies from those at risk of major cardiac toxicities. There are few reports of biomarkers for the prediction of, or early detection of IrAEs in general. These include changes in the expression of cytokines/chemokines, cellular markers, autoantibodies, and genes. There is unfortunately no report describing markers selective for cardiac specific IrAEs (124). Currently, putative biomarkers for cardiac-specific IrAEs are limited to the serum levels of proteins such as cardiac troponin (cTn), and myoglobin but these are largely not selective for ICI IrAEs and not supported by extensive clinical validation.

### 7.1 Non cardiac biomarkers

**Peripheral blood count (PBC)**: The indices and absolute values of peripheral blood components such as leukocytes, neutrophils and lymphocytes and platelets have been well established as prognostic markers for ICI responses and outcomes in several cancers (125). Several studies have also demonstrated PBC indices as predictive of ICI toxicities. For example, a recent retrospective study showed that an absolute lymphocyte count >820 at 2 weeks following nivolumab initiation predicts the early onset of irAEs during in a 6-week study period (126). Routinely available absolute lymphocyte count may therefore be useful for identifying patients at risk of early onset of ICI irAEs (126). Prospective studies are warranted in this area.

**Cytokines/Chemokines:** Lim and colleagues recently profiled the expression of 65 cytokines in 98 patients with melanoma treated with PD-1 inhibitors alone or in combination with anti-CTLA-4 (127). Cytokine expression was found to strongly correlate with irAEs warranting discontinuation of treatment and administration of high-dose steroids. Eleven cytokines significantly upregulated in patients with severe irAEs were integrated into a single toxicity score known as the CYTOX (cytokine toxicity) score. The most predictive cytokines for ICI toxicities include G-CSF, GM-CSF, Fractalkine, FGF-2, IFN-aplha2, IL12p70, IL1a, IL1B, IL1RA, IL2, and IL13 (127). The predictive utility of CYTOX score was confirmed in an independent validation cohort of 49 patients treated with combination anti-PD-1 and anti-CTLA-4 (127). The utility of CYTOX in predicting cardiac specific IrAEs has yet to be validated.

### 7.2 Cardiac specific biomarkers

**Cardiac Troponins:** Cardiac troponin T (cTnT) and cardiac troponin I (cTnI) are expressed exclusively in the myocardium. They are elevated in 84% to 94% of patients with ICI cardiotoxicity (including subclinical toxicities) (59, 105, 121). cTnl is often preferred for cardiac IrAEs as cTnT and other cardiac biomarkers such as CPK, BNP and/or proBNP may be elevated in patients with concurrent pathologies associated with ICI. For example, CPK is elevated in myositis which can be immune mediated. (cTn) are released after cardiomyocytes damage induced by various mechanisms such as ischemia, inflammation, oxidative stress, or apoptosis. Several studies have reported increased risk of ICI induced cardiac irAEs in patients with elevated pre-treatment troponin. Mahmood and colleagues compared the data of patients with and without myocarditis after ICI treatment and found a four-fold increase in the risk of cardiac irAEs for patients with troponin T (cTnT) ≥ 1.5 ng/ml (116, 121, 128). Another retrospective cohort study demonstrated a seven fold risk of cardiac IrAEs in patient receiving ICIs with baseline troponin >0.01 ng/ml (HR: 7.27; 95% CI: 2.72 to 19.43; p < 0.001) (129). Although currently not recommended by the ASCO updated guideline, there is a growing consensus to perform baseline troponin measurements prior to initiating ICIs The Heart Failure Association Cardio-Oncology Study Group and the International Cardio-Oncology Society risk stratification guidelines for anticancer therapies recommends pretreatment troponin determination (130).

**Cardiac Auto-antibodies:** Okazaki and colleagues showed that dilated cardiomyopathy in PD-1 deficient mice is associated with their production of high titer autoantibody against cardiac troponin I (71, 72). Cardiac troponin I auto antibodies have yet to be validated as a biomarker for cardiac irAEs.

**Brain-type natriuretic peptide (BNP**): BNP and N-terminal pro-brain natriuretic peptide (NT-proBNP) are standard biomarkers used in clinical practice for the diagnosis and management of heart failure. However, conclusions regarding the role of natriuretic peptides for the risk analysis and diagnosis of ICI cardiotoxicity remain undefined. A retrospective studies demonstrates an increased risk of ICI adverse event at B-type natriuretic peptide (BNP) >100 pg/ml (HR: 2.65; 95% CI: 1.01 to 6.92; p = 0.047) (129).

## 8 Roadmap to overcoming ICI-induced cardiac irAEs

### 8.1 Development and validating of prognostic biomarkers for cardiac irAEs

As discussed, existing biomarkers for ICI cardiac irAEs have relatively limited clinical data and/or lack extensive validation. Biomarkers that are appropriately sensitive and specific to therapy-induced injury could find applications in long-term post therapy management, subclinical toxicity detection, and pre therapy risk stratification for ICI therapy (131). Future biomarkers for cardiac irAEs would be sensitive enough to detect subclinical conditions but specific enough not to arise from the cancer itself. Several have been proposed or are under investigation. Modern capabilities in systems biology and genetics have enabled novel techniques like high-through sensitive bioassays and multiomics approaches (131). Currently proposed blood biomarkers include high-sensitivity troponin levels (hs-TnI), microRNAs, C-reactive protein, myeloperoxidase, galectin 3, interleukin family molecules including ST2, matrix metalloproteinase, placental growth factor (PlGF), growth differentiation factor 15, peripheral blood mononuclear cell gene expression profile, and human heart-type fatty acid-binding protein (132) (133). Many of these biomarkers are nonspecific to ICI as they have been detected at elevated levels following other systemic therapies and cardiac radiotherapy (133, 134). Nevertheless, pre-treatment hs-Tnl levels (detected using a modification of the fourth-generation cTnT assay) at a cut-off of 14ng/L have been demonstrated to predict cardiovascular endpoints and the progression of cardiac involvement in patients receiving Nivolumab (135). It is notable in this regard that the Stanford Cancer Institute has recently implemented surveillance for ICI-associated myocarditis with hs-TnI assay (136, 137). Another predictive measure for cardiac irAEs severity following ICI therapy may be the levels of certain microRNAs. Pre-clinical studies have demonstrated an increased frequency and severity of irAEs in murine models deficient in miR-146a and studies of humans subjects have demonstrated an increased risk of severe irAEs in patients on anti PD-1 therapy who have a single nucleotide polymorphism (SNP) in miR-146a (138). MiR-34a is a critical regulator of myocardial physiology that increases with age and has been associated with cardiac senescence and dysfunction. Through a variety of effects on the NF-κB and KLF4 signaling pathways miR-34a also modulates T cell and macrophage functions such that elevated levels may predispose patients to ICI-related cardiac toxicities (67, 139–141). Further studies of baseline and post-treatment levels of these and other miRs are required to substantiate the likelihood that these may have utility as prognostic biomarkers for ICI cardiac irAEs.

Besides circulating biomarkers, functional and MRI imaging markers have also been proposed to predict ICI toxicities. Cardiac PET scans entail exposure to ionizing radiation, but studies suggest they may be indicated for measuring long-term ICI effects on the heart (142) . Advanced radioscopic imaging techniques may also evaluate myocardial and vascular changes at the molecular level (142). A recent retrospective study identified septal late gadolinium enhancement as a possible predictor of cardiac event in patients receiving ICIs (143). It will be essential to contextualize any findings from circulatory and imaging biomarkers with the specific mechanism of IrAEs. For example, ICI-associated myocarditis biomarkers may detect between the different phenotypes of myocarditis; lymphocytic myocarditis is facilitated by proinflammatory T_H_17 cells and CCR5, and giant cell myocarditis is thought to originate from the autoantigen-triggered immunoproteasome, leading to CD4^+^ T cell recruitment and differentiation into T_H_1 and T_H_17 cells (64). Specific biomarkers along these immunological axes may be candidates for novel biomarkers of ICI-specific cardiac irAEs.

### 8.2 Utilization of immune checkpoint inhibitors with reduced cardiotoxicity

A shift in focus to research and development of novel ICIs which target antigens that are not shared amongst both the myocardium and tumor in question, unlike the current targets PD-1, PD-L1, CTLA-4, and LAG-3 may limit inflammatory reactions against cardiomyocytes. New drugs under investigation include anti-TIM-3 (T cell immunoglobulin and mucin-containing protein 3), anti-VISTA (V-domain Ig suppressor of T cell activation), anti-TIGIT, and anti-BTLA antibodies (144). These targets have each been shown to restore antitumor immunologic response in preclinical studies, and they are currently under study in humans (144). Cardiotoxicity of these agent are currently unknown. It is of utmost importance that these ongoing human studies prioritize the assessment of adverse event including cardiac toxicities in addition to cancer outcomes

### 8.3 Novel prophylaxis and therapies for cardiac irAEs

Current strategy for management of for ICI induced irAEs are empirical as no studies have specifically addressed the issue. There is potential for further development of anti-inflammatory agents that are specific to the myocardium, which may be administered prophylactically or in combination with current ICIs to avert cardiac irAEs. Immune modulators which have been shown in case reports or small case series to be effective in reversing near-lethal ICI-myocarditis. Drugs which have been investigated include tocilizumab (IL-6R antibody) (145), alemtuzumab (anti-CD52) (17) (146), abatacept (CTLA-4 agonist) (147), ruxolitinib (JAK inhibitor) (148), infliximab (TNFα antibody) (149), tofacitinib (JAK inhibitor) (150), mycophenolate mofitil (151), and antithymocyte globulin (152), and IV immunoglobulin. (153) However, the effectiveness of these therapies in ICI induced cardiac irAEs is unclear and they are therefore only reserved for patients with poor responses to corticosteroids. Further studies are needed to better understand the clinical indication and safe dosage for these drugs in patients with cardiac irAEs (154). For example, the ongoing ATRIUM trial (Clinicaltrial.gov NCT05335928) is being carried out to assess whether abatacept therapy, as compared to placebo, is associated with a reduction in major adverse cardiac events (MACE) among participants hospitalized for ICI-induced myocarditis.

The recent findings that anti-PD-1 therapy induces metabolic dysregulation associated with cardiac dysfunction raises the prospect of metabolic intervention for cardiac irAEs (70, 155) (81). Increased expression of TNFα is a notable downstream effect of anti-PD1 therapy which can lead to myocardial dysfunction *via* suppression of L-type calcium channel and ryanodine receptor-2 activities in addition to its pro-inflammatory activities. Michel and colleagues demonstrated that TNFα blockade could avert the associated subclinical manifestation of cardiac dysfunction due to anti-PD1 therapy in mice models without attenuating its anti-cancer efficacy. They hypothesize TNFα blockade may serve as a novel cardioprotective treatment against ICI therapy (70, 81, 155, 156) . Such an outcome may be expected as inflammatory mechanisms driven by TNFα are likely to have responsibility for ICI-induced cardiotoxicity but be less important for T cell-mediated anti-tumor immunity.

## 9 Conclusion

In conclusion, some advances have been made in elucidating the pathologic mechanisms of ICI-associated cardiac irAEs in recent years. Histopathologic grading criteria with diagnostic and prognostic values have been developed but are yet to be standardized and universally adopted. Potential strategies for mitigating ICI-associated irAEs include: Developing and validating predictive biomarkers; developing and utilizing less cardiotoxic ICIs; administering prophylactically or in combination with ICIs to avert cardiac irAEs; and prospective trials of known anti-inflammatory agents with therapeutic benefit in patients with cardiac irAEs.

## Author contributions

OI researched data for the article, made substantial contributions to discussions of the content and wrote the article. NN, YS, MN, and SP made substantial contribution to the writing and illustration of figures. ES, DS, DH, and BL made substantial contributions to discussions of the content and reviewed and/or edited the manuscript before submission. All authors contributed to the article and approved the submitted version.

## Funding

BL National Cancer Institute NCI 1R01 CA252484-01.

## Conflict of interest

The authors declare that the research was conducted in the absence of any commercial or financial relationships that could be construed as a potential conflict of interest.

## Publisher’s note

All claims expressed in this article are solely those of the authors and do not necessarily represent those of their affiliated organizations, or those of the publisher, the editors and the reviewers. Any product that may be evaluated in this article, or claim that may be made by its manufacturer, is not guaranteed or endorsed by the publisher.
